# Shortening and re-lengthening versus bone transport for the treatment of distal tibial periarticular post-traumatic defects

**DOI:** 10.1038/s41598-022-20760-0

**Published:** 2022-09-29

**Authors:** Qiang Huang, Teng Ma, Cheng Ren, YiBo Xu, Ming Li, Qian Wang, Yao Lu, Zhong Li, Kun Zhang

**Affiliations:** Department of Orthopedic Surgery, Hong Hui Hospital, Xi’an Jiaotong University College of Medicine, Xi’an, 710054 Shaanxi China

**Keywords:** Bone, Infection

## Abstract

In the present study, we presented our experience with a new modified technique of shortening and re-lengthening using a monolateral external frame combined with a calcaneal intramedullary nail and compared it with the bone transport technique for the treatment of distal tibial periarticular post-traumatic defects. Forty-one patients were retrospectively analyzed. Among them 19 were treated using our modified shortening and re-lengthening technique (MSR group) and 22 by bone transport (BT group). The difference in external fixation time (EFT), external fixation index (EFI), self-rating anxiety scale (SAS), and complications were compared between the two groups. The mean EFT was 3.4 ± 0.6 months in the MSR group and 7.5 ± 1.4 months in the BT group; the EFI was 0.57 ± 0.06 month/cm and 1.32 ± 0.23 month/cm, respectively. The EFT, EFI, and SAS scores were significantly lower in the MSR group than in the BT Group (*p* < 0.05). The mean number of complications per patient in the BT group was nearly 2.4 times that of the MSR group (*p* < 0.05). Our modified shortening and re-lengthening technique reduced the EFI and complication incidence compared to the bone transport technique. Therefore, patients with distal tibial periarticular post-traumatic defects can achieve great satisfaction with this new technique.

## Introduction

The number of patients suffering from severe open fractures, osteomyelitis, or nonunion of the distal tibia has increased. After radical debridement, large segmental periarticular defects often occur at the distal tibia. The reconstruction of such defects is complex, and the complication incidence is high, comprehending a severe challenge for trauma surgeons^1^. The Ilizarov technique has been widely used to treat large segmental bone defects. Distraction osteogenesis can effectively solve the problem of insufficient donor area for such defects, nonunion of bone grafts, and limb shortening^2^. However, one of the main disadvantages of the Ilizarov technique is the long consolidation period, which is nearly two to three times of the transport period. This delay leads to a significantly increased risk of complications, such as axial deviation, pin-tract infection, foot drop, joint stiffness, and re-fracture at the regenerate site^3^. Moreover, the long-term wearing of an external fixator is very inconvenient to patients and can make them anxious and depressed^4^.

Several modified techniques have been used to repair large segmental bone defects, such as bone transport over a nail or plate, shortening and re-lengthening^5–9^. In our previous study, we successfully used the hybrid transport technique to treat large segmental tibial defects^5^. We found that bone transport over a tibial intramedullary nail or a locking plate could significantly shorten the time in the external fixator and reduce the incidence of postoperative complications. However, since a tibial intramedullary nail can not provide effective fixation for distal tibial defects, this technique is unsuitable for patients with distal tibial periarticular defects. Shortening and re-lengthening has also been used in patients with large segmental bone defects of the tibia and femur^6–8^ to shorten the limb through acute or chronic traction, temporarily eliminate the bone defect segment, and obtain the same length as the healthy side through gradual traction and lengthening. Nevertheless, whether it can reduce the EFI and complication incidence remains controversial. Until now, shortening and re-lengthening technique has not been reported in patients with distal tibial periarticular defects.

Our team has developed and used a novel technique for several years that represents the modification of the shortening and re-lengthening technique to overcome the disadvantages of the bone transport technique. Herein, we aimed to introduce our clinical experience with the new technique of shortening and re-lengthening using a monolateral external frame combined with a calcaneal intramedullary nail and to compare it with the classic bone transport technique for the treatment of distal tibial periarticular defects secondary to radical debridement of the distal tibia.

## Materials and methods

### Patients

The ethics committee of the Xi’an Hong Hui hospital approved this study (No. 202208008), and written informed consent was obtained from all patients. All methods were carried out following relevant guidelines and regulations^10^. Clinical and imaging data of 41 patients with distal tibial periarticular defects treated in our institution from January 2012 to January 2018 were retrospectively analyzed. The inclusion criteria were: (1) Patients over 18 years; (2) Patients with distal tibial periarticular post-traumatic defects. “Distal tibial periarticular defects” meant bone defects within the distal third of the tibia and the involvement of the ankle joint; (3) Bone defects were between 2 to 10 cm; (4) Anatomical reconstruction of the ankle was not possible; (5) Clinical and imaging data were complete. The exclusion criteria were: (1) Patients younger than 18 years; (2) Patients suffering from tibial defects without involving the ankle joint; (3) Patients with severe medical diseases unable to tolerate anesthesia and surgery; (4) Patients with severe limb damage and forced amputation; (5) Patients with incomplete clinical data. Finally, 19 patients were treated using our modified shortening and re-lengthening technique (MSR group) and 22 by bone transport (BT group).

### Preoperative treatment

The inflammatory indexes were detected before operations, and the wound secretion was taken for bacteriological culture and drug sensitivity test. At the same time, full-length X-ray images of the tibia, X-ray, and CT of the ankle joint were performed.

### Surgical procedures of the MSR group

After radical debridement, the foot was placed in the neutral position. Both ends of bone defects at the distal tibia were trimmed and leveled. Rotation and varus deformity were eliminated before temporary fixation. The injured limb was initially shortened to make the bone defect ends contact each other. At this time, circulation was closely observed. If the acute shortening could not be completed, the injured limb was first shortened by 4–5 cm, then 2–3 mm every day for the next two weeks until the broken ends contacted each other. Soft tissue reconstruction would become relatively easy after shortening. Soft tissue defects were repaired according to the “ladder principle”; if it can be directly sutured, skin graft should be avoided; if skin graft could be used, flap surgery was unnecessary.

Bone lengthening was performed when the soft tissues healed well and the inflammatory indexes returned to normal. After successful anesthesia, the injured limb was placed in the neutral position. The entry point of the calcaneal intramedullary nail was confirmed at the plantar area under C-arm fluoroscopic guidance. The guide pin was inserted first. The medullary cavity was appropriately expanded as needed. Then, an appropriate calcaneal intramedullary nail was inserted. The diameter of the nail was 1 mm thinner than the narrowest part of the tibial medullary cavity. The tail of the nail could reach the proximal tibia. After confirming that the axis and rotation of the distal tibia were good, the head of the calcaneal intramedullary nail was locked. Next, the monolateral external fixator was installed. The distal and proximal ends of the lengthening frame were fixed by Schanz nails. Proximal Schanz nails were inserted from the medial side of the proximal tibia. Distal Schanz nails were inserted from the inner side of the calcaneus and talus. Low energy osteotomy was performed with a wire saw at the metaphysis of the distal tibia. Finally, the wound was rinsed and sutured.

**Figure 1:**
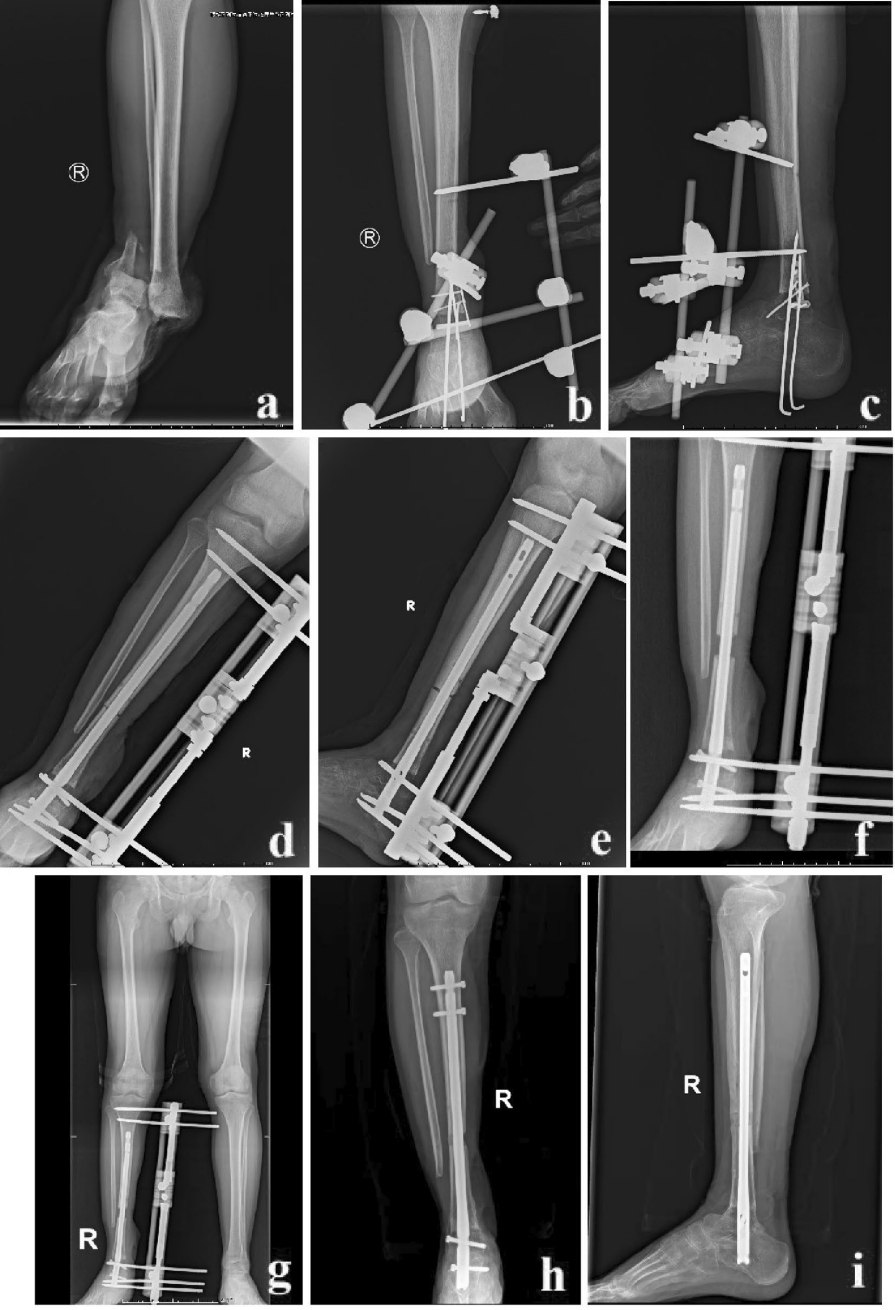
A 20-year-old male was successfully treated by the MSR technique. (**a**) The patient suffered from a severe open distal tibial fracture; (**b**) and (**c**) The distal tibial bone defects were observed after radical debridement. Thus, acute shortening and temporary fixation were performed; (**d**–**g**) The MSR technique was used to restore limb length; (**h**) and (**i**) Half a year after removing the lengthening frame, the consolidation of the regenerate segment was good. MSR: modified shortening and re-lengthening.

Bone lengthening started one week later. The initial speed was 1.0 mm/day, completed in four to six times. The bone lengthening stopped when both lower limbs were equal in length. At this time, the lengthening frame was removed. The tail of the calcaneal intramedullary nail was locked by inserting two locking screws. A typical case is shown in Fig. 1.

### Surgical procedures of the BT group

**Figure 2:**
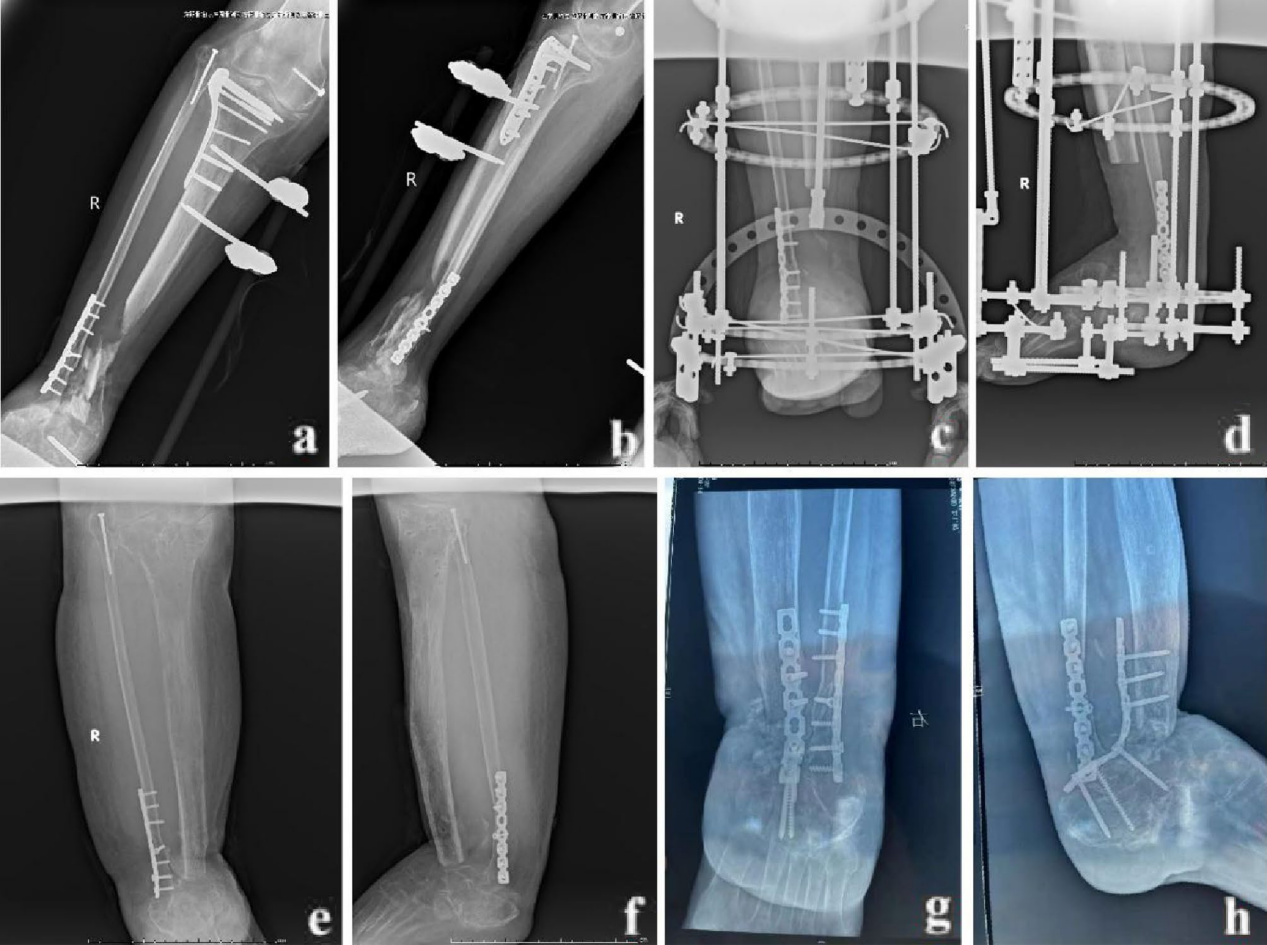
A 25-year-old male was treated by the classic BT technique. (**a**) and (**b**) The patient suffered from osteomyelitis and bone loss of the distal tibia; (**c**) and (**d**) The classic BT technique was performed after radical debridement; (**e**) and (**f**) Although the transport and consolidation were successfully completed, docking site nonunion occurred; (**g**) and (**h**): The docking site healed after bone grafting and internal fixation. BT: bone transport.

The classic method of bone transport was performed as previously described^5^. After the infection was controlled and the soft tissues healed, the classic bone transport was performed. The lower leg was maintained in the center of the Ilizarov frame, fixed with several Kirschner wires parallel to the knee and ankle joint plane. The proximal tibial osteotomy was performed, Kirschner wires were inserted to fix the transport segment, and the alignment and rotation were adjusted under C-arm fluoroscopic guidance to ensure no axial deviation. Then, the wounds were washed and sutured. The transport frame was adjusted for bone transport one week after the operation. During the bone transport process, the condition of nerves and circulation was closely monitored. The transport frame was removed after the docking site firmly healed, and the new callus was completely consolidated. A typical case is shown in Fig. 2.

### Postoperative treatment

All patients were treated with sensitive antibiotics for six weeks according to the drug sensitivity test results. Antibiotics were adjusted in time, and inflammatory indexes were regularly evaluated. Patients were instructed to perform appropriate passive and active exercises after the operation. Patients were followed once every two weeks during the bone transport/lengthening process. Pin-tract nursing was regularly carried out to prevent infection. The weight-bearing and walking time of the injured limb were determined by X-ray images, symptoms, and signs.

### Observation indexes

The external fixation time (EFT), external fixation index (EFI), and self-rating anxiety scale (SAS) scores^11^ were monitored during the follow-up period. Bone defect healing was evaluated by the Paley score^12^ as follows: (1) bone healing; (2) no recurrence of infection; (3) limb deformity less than 7°; (4) unequal length of limbs < 2.5 cm. The grade was determined as: excellent: good bone healing + (2) ~ (4) indexes for all items; good: good bone healing + (2) ~ (4) indexes for two items; fair: good bone healing + (2) ~ (4) index for one item; poor: bone nonunion or refracture + (2) ~ (4) indexes for no items. Complications were categorized according to the Paley classification^13^, including problems, obstacles, and sequelae. Ankle and foot function was assessed by the modified Kitaoka score, which included the activity level, walking distance, limp, ankle flexion/extension range, and pain^14^. The maximum value was 80 points.

### Statistical analysis

SPSS 24.0 software was used to process data and perform statistical analyses by the same authors. Measurement data are expressed as mean ± standard deviation (SD) and were analyzed by unpaired t-tests. Count data were analyzed using the χ2 test. A *p* < 0.05 was considered statistically significant.

### Consent to participate/Consent to publish

All patients or their family members have signed the informed consent before surgery and provided the consent to publish and report individual clinical data.

## Results

### Demographic information

Herein, we enrolled 41 patients with distal tibial periarticular defects, including 32 males and 9 females, with ages between 20 and 59 years. The mean age was 36 ± 11 years in the MSR group and 34 ± 12 in the BT group. There were eight cases with at least one medical disease in the MSR group and ten in the BT group. In the MSR group, 13 cases suffered from traffic accidents, 4 had heavy pound injury, and 2 from falling height. Meanwhile, the BT group comprised 16 cases suffering from traffic accidents, 3 from heavy pound injury, and 3 from falling height. The initial injury was open in 12 cases for the MSR group and 14 for the BT group. There were 8 cases for the left leg and 11 for the right in the MSR group, and 9 for the left and 13 for the right leg in the BT group. The follow-up time was 24–39 months, with an average of 27 ± 5 months for the MSR group and 28 ± 5 months for the BT group. The two groups had no significant demographic data differences (*p* > 0.05, Table 1).

**Table 1:** Demographic data of two groups.

Variable	MSR group (n = 19)	BT group (n = 22)	*P* value
Age (year)	36 ± 11	34 ± 12	0.583
**Gender**			0.803
Male	15	17	
Female	4	5	
**Etiology**			
Traffic accident	13	16	0.763
Falling height	2	3	0.861
Heavy pound injury	4	3	0.831
**Initial injury**			0.975
Open	12	14	
Closed	7	8	
**Side**			0.938
Left	8	9	
Right	11	13	
**Medical disease**			0.829
None	11	12	
At least one kind	8	10	
Bone defect length (cm)	6.0 ± 1.3	5.7 ± 1.5	0.501
Follow-up (month)	27 ± 5	28 ± 5	0.529

MSR stands for modified shortening and re-lengthening. BT stands for bone transport.

### Comparison of outcome parameters between the two groups

The results for EFT, EFI, SAS, Paley, and Kitaoka scores are presented in Table 2. The mean EFT was 3.4 ± 0.6 months in the MSR group and 7.5 ± 1.4 months in the BT group. The mean EFI was 0.57 ± 0.06 month/cm and 1.32 ± 0.23 month/cm in the MSR and BT groups, respectively. Regarding the SAS score, the MSR group included 15 patients with no anxiety, 3 with mild anxiety, and 1 with moderate anxiety. The BT group comprised 6 patients with no anxiety, 10 with mild anxiety, and 6 with moderate anxiety. The EFT, EFI, and SAS scores were significantly lower in the MSR group than in the BT group (*p* < 0.05). Moreover, all patients with distal tibial periarticular defects achieved healing, and no difference was observed between the two groups (*p* > 0.05). The bone defect healing score in the MSR group was excellent for 17 patients, good for 1, and fair for 1. In the BT group, it was excellent for 15 cases, good for 4, and fair for 3. The mean modified Kitaoka score was 62.5 ± 6.3 points in the MSR group and 56.5 ± 7.9 points in the BT group, with a significant difference between the two groups (*p* < 0.05).

**Table 2:** Comparison of outcome parameters between the two groups.

Variable	MSR group (n = 19)	BT group (n = 22)	*P* value
EFT (month)	3.4 ± 0.6	7.5 ± 1.4	0.001
EFI (month/cm)	0.57 ± 0.06	1.32 ± 0.23	0.001
**SAS score**			0.001
No anxiety	15	6	
Mild anxiety	3	10	
Moderate anxiety	1	6	
**Bone defect healing score**			
Excellent	17	15	0.206
Good	1	4	0.434
Fair	1	3	0.709
Modified Kitaoka score	62.5 ± 6.3	56.5 ± 7.9	0.010

MSR stands for modified shortening and re-lengthening. BT stands for bone transport. EFT stands for external fixation time. EFI stands for external fixation index. SAS stands for self-rating anxiety scale.

### Complications between the two groups

Complications occurred in both groups and were categorized according to the classification of Paley, including problems, obstacles, and sequelae (Table 3). In the BT group, 23 problems were detected, including 12 Grade-II pin-tract infections, 5 delayed maturations of the regenerate site, 4 transient losses of knee movement, and 2 angulations at the regenerate site. Patients with Grade-II pin-tract infection received oral antibiotics and regular dressing. Those with delayed maturation of the regenerate site were closely monitored. Patients with transient loss of knee movement were instructed to perform appropriate functional exercises, and two received manual release under oral painkillers. Meanwhile, 18 obstacles were observed in the BT group, including 5 Grade-III pin-tract infections, 8 docking site nonunions, 3 axial deviations, and 2 soft tissue invaginations. Grade-III pin-tract infections were controlled by removing the infected pins and re-inserting another one. Six patients with docking site nonunion were managed by bone grafting and internal fixation, while the other two received only bone grafting. The axial deviation was corrected using surgery to adjust the external frame. Patients with soft tissue invagination received a surgical release. Seven sequelae were detected in the BT group, including 4 foot drops and 3 varus or valgus deformities. These patients rejected suggestions for further corrections.

**Table 3:** Comparison of complications between the two groups.

Complications	Number of complications (MSR group )	Number of complications (BT group )	*P* value
**Problem**			
Grade-II pin-tract infection	4	12	
Delayed maturation of regenerate site	2	5	
Transient loss of knee movement	2	4	
Angulation at regenerate site		2	
**Obstacles**			
Grade-III pin-tract infection	2	5	
Docking site nonunion	2	8	
Axial deviation		3	
Soft tissue invagination		2	
**Sequelae**			
Foot drop	1	4	
Varus or valgus deformity	1	3	
Total (Number of complications)	15	48	
Number of complications per patient (mean)	0.9 ± 0.6	2.2 ± 0.7	0.001

MSR stands for modified shortening and re-lengthening. BT stands for bone transport.

In the MSR group, eight problems were observed, comprising 4 Grade-II pin-tract infections, 2 delayed maturations of the regenerate site, and 2 transient losses of knee movement. Additionally, four obstacles were detected, 2 Grade-III pin-tract infections and 2 docking site nonunion. Two patients developed sequelae, including one foot drop and one varus deformity. The management of these complications was similar to the BT group. The mean number of complications per patient in the BT group was nearly 2.4 times higher than in the MSR group (*p* < 0.05).

## Discussion

Distal tibial periarticular defects are complex injuries encountered by trauma surgeons as it is necessary to deal with large segmental bone defects and ankle stability simultaneously. These patients are often secondary to infection after internal fixation or serious open fractures. Thus, patients and their families suffer from a great financial, physical, and mental burden. Even if experienced professionals handle them, various complications and dysfunctions may still occur. Therefore, the long-term prognosis of these patients can be poor.

Treatment methods for large segmental bone defects include autologous bone transplantation, vascularized fibular transplantation, the induced membrane technique, and the Ilizarov technique^2,15–18^. The Ilizarov technique is a classic method for patients with large segmental bone defects and has saved many limbs on the verge of amputation. This technique has a high final success rate, presenting a final bone defect healing rate of over 90%^19^. However, the main disadvantage of this technique is the long-lasting consolidation period and many external frame-related complications^2,12,18^. Several scholars have explored different modified techniques to reduce the EFI and related complications, such as shortening and re-lengthening and bone transport over a nail^5–9^. For example, the EFI is between 1.0 and 1.9 months/cm by bone transport with Ilizarov external frame^1–4,6,8^. Combined with an intramedullary nail, the time in the external fixator can be effectively shortened, and the EFI can reach 0.45–0.87 months/cm. Liodakis et al.^20^ also found that bone transport over an intramedullary nail can reduce the time in an external fixator and deformity incidence. When combined with an intramedullary nail, the external fixator can be removed early, and the EFI can be reduced, consistent with our previous research^5^.

Shortening and re-lengthening has also been used to treat large segmental bone defects^6–8^. Several scholars compared the clinical outcomes between shortening and re-lengthening and classical bone transport. Sigmund et al.^6^ used these techniques to treat 47 patients with complex tibial infected non-unions and osteomyelitis. Their results showed that the bone transport group demonstrated a higher rate of unplanned surgeries, especially docking site revisions. On the other hand, no significance was detected for the EFI between the two groups. Additionally, Thakeb et al.^7^ included 50 patients in a nonrandomized prospective study. The incidence of complications and ASAMI scores for bone and functions were not statistically significant between the two groups. Nevertheless, the shortening and re-lengthening technique had a significantly lower EFI. In Tetsworth’s research^8^, the shortening and re-lengthening group had a lower complication incidence and a slightly better radiographic outcome. As the inclusion criteria of the above studies differed, there was a selection bias. Trauma surgeons also preferred bone transport and shortening/re-lengthening. Overall, the shortening and re-lengthening technique is slightly better than bone transport for 3 to 10 cm of bone loss. Sen et al.^21^ presented their experience with a new modified technique of shortening and re-lengthening using a monolateral external fixator combined with a retrograde intramedullary nail to manage infected non-unions of the distal femur with bone loss. Their modified shortening and re-lengthening technique conferred greater patient satisfaction because of shorter EFI.

In the present study, we combined a calcaneal intramedullary nail with a shortening and re-lengthening technique to treat distal tibial periarticular post-traumatic defects. Our modified technique could simultaneously deal with the problems of large segmental bone defects and ankle stability. As a new type of internal fixation for ankle fusion, the calcaneal intramedullary nail has been more widely used recently and achieved good results^22–24^. Biz et al. reported that minimally invasive surgery for tibiotalocalcaneal arthrodesis with the calcaneal intramedullary nail results in fusion of the articulation with a low complication rate^24^. It belongs to central and multi-point fixation, which is conducive to early weight-bearing exercise. However, it has not been used in repairing large segmental bone defects. We have been working on the “lengthening over the calcaneal intramedullary nail” technique for several years. The mean EFI in our study was 0.57 ± 0.06 month/cm by this modified technique, similar to the above results of the Ilizarov technique combined with a tibial or femoral intramedullary nail. We could remove the external frame early for calcaneal intramedullary nails. The calcaneal intramedullary nail provided mechanical stability during the callus consolidation period, and patients felt comfortable without the external frame, which was helpful for the whole treatment process. In our study, the SAS score was significantly lower in the MSR group than in the BT group. Thus, the modified technique significantly released patients’ physical and mental pressure.

Furthermore, we showed that the mean number of complications per patient was significantly lower in the MSR group than in the BT group because the lengthening frame could be removed early, and the complications caused by the external frame were significantly reduced. Regarding the details of complications, the most significant differences between the two groups were detected for the incidence of pin-tract infection and docking site nonunion. In the MSR group, six patients suffered from pin-tract infection, including 4 Grade-II and 2 Grade-III cases. On the other hand, in the BT group, 17 patients had pin-tract infections, comprising 12 Grade-II and 5 Grade-III cases. The BT group had 2.8 times more infections than the MSR group. This difference might be caused by the significantly different EFT between the two groups. Additionally, the BT group (eight cases) had four times more docking site nonunion than the MSR group (two cases). The main reason might be that soft tissues were embedded in the defect segment and the bone ends hardened during bone transport. Moreover, the incidence of sequelae in the MSR group (2 cases, 11%) was significantly lower than in the BT group (7 cases, 32%) because, in the MSR group, the bone defect ends were aligned and could heal early, and both bone defect ends were more stable under the fixation of internal and external devices, which was conducive to ankle fusion. However, in the BT group, soft tissue embedding could easily lead to delayed healing or even docking site nonunion, and the bone ends could not obtain absolute stability until healing, which might be the root cause of ankle deformity.

Our current study also has some limitations. First, the number of cases was limited, and the follow-up time was short. Second, this research was a retrospective case–control study. Additionally, the chief surgeon might have a preference when deciding which method to apply for distal tibial periarticular post-traumatic defects, which might result in selection bias. Nevertheless, this selection bias did not significantly interfere with our results. Third, bone transport on large defects could have docking site problems and soft tissue invagination, but it can be managed along with the conversion of Ilizarov into internal fixation, as we knew in lengthening and than plating or nailing technique, soon after the length achieved. It also shortened the external fixation index. We did not know whether our modified shortening and re-lengthening technique is superior to the lengthening and than internal fixation technique. In further studies, we will try to compare our modified technique with bone transport and then internal fixation technique. These deficiencies will be improved in further research. Although there was no infection recurrence in the enrolled patients, we were still worried about this complication. Therefore, radical debridement and careful protection of local soft tissues cannot be overemphasized.

## Conclusion

In summary, our modified shortening and re-lengthening technique reduced the EFI and complication incidence, especially pin-tract infection and docking site nonunion, compared to the classic BT technique. Additionally, the modified technique displayed better limb functions than the BT technique. In the future, we will conduct prospective randomized controlled research to corroborate these conclusions.

## Data Availability

The datasets analyzed during the current study are available from the corresponding author upon reasonable request.

## References

[1] Tong K, Zhong Z, Peng Y, Lin CG, Wang G (2017). Masquelet technique versus Ilizarov bone transport for reconstruction of lower extremity bone defects following posttraumatic osteomyelitis. Injury.

[2] Aktuglu K, Erol K, Vahabi A (2019). Ilizarov bone transport and treatment of critical-sized tibial bone defects: A narrative review. J. Orthop. Traumatol..

[3] Biz C, Crimì A, Fantoni I, Vigo M, Iacobellis C, Ruggieri P (2021). Functional outcome and complications after treatment of comminuted tibial fractures or deformities using Ilizarov bone transport: A single-center study at 15- to 30-year follow-up. Arch. Orthop. Trauma Surg..

[4] Huang Q (2022). Bone transport combined with bone graft and internal fixation versus simple bone transport in the treatment of large bone defects of lower limbs after trauma. BMC Musculoskelet. Disord..

[5] Huang Q (2021). Antibiotic calcium sulfate-loaded hybrid transport versus traditional Ilizarov bone transport in the treatment of large tibial defects after trauma. J. Orthop. Surg. Res..

[6] Sigmund IK, Ferguson J, Govaert G, Stubbs D, McNally MA (2020). Comparison of Ilizarov bifocal, acute shortening and relengthening with bone transport in the treatment of infected, segmental defects of the tibia. J. Clin. Med..

[7] Thakeb MF (2019). Bifocal compression–distraction for combined bone and soft-tissue defects in post-traumatic tibial nonunion. J. Orthop. Trauma.

[8] Tetsworth K (2017). Bone transport versus acute shortening for the management of infected tibial non-unions with bone defects. Injury.

[9] Biz C, Iacobellis C (2014). Nailing treatment in bone transport complications. Strateg. Trauma Limb Reconstr..

[10] Padulo J, Oliva F, Frizziero A, Maffulli N (2013). Muscle, Ligaments and Tendons Journal. Basic principles and recommendations in clinical and field science research. Muscles Ligaments Tendons J..

[11] Zung WW (1971). A rating instrument for anxiety disorders. Psychosomatics.

[12] Paley D, Maar DC (2000). Ilizarov bone transport treatment for tibial defects. J. Orthop. Trauma.

[13] Paley D (1990). Problems, obstacles, and complications of limb lengthening by the Ilizarov technique. Clin. Orthop. Relat. Res..

[14] Kitaoka HB, Alexander IJ, Adelaar RS, Nunley JA, Myerson MS, Sanders M (1994). Clinical rating system for the ankle-hindfoot, midfoot, hallux, and lesser toes. Foot Ankle.

[15] Ronga M (2019). Induced membrane technique for the treatment of severe acute tibial bone loss: Preliminary experience at medium-term follow-up. Int. Orthop..

[16] Antonini A (2019). Bone defect management with vascularized fibular grafts in the treatment of grade III-V osteomyelitis. Handchir. Mikrochir. Plast. Chir..

[17] Masquelet AC, Kishi T, Benko PE (2019). Very long-term results of post-traumatic bone defect reconstruction by the induced membrane technique. Orthop. Traumatol. Surg. Res..

[18] Paley D (1989). Ilizarov treatment of tibial nonunions with bone loss. Clin. Orthop. Relat. Res..

[19] Oh CW (2013). Bone transport with an external fixator and a locking plate for segmental tibial defects. Bone Jt. J..

[20] Liodakis E (2011). Comparison of 39 post-traumatic tibia bone transports performed with and without the use of an intramedullary rod: The long-term outcomes. Int. Orthop..

[21] Sen C (2019). Acute shortening versus bone transport for the treatment of infected femur non-unions with bone defects. Injury.

[22] Steele JR, Lazarides AL, DeOrio JK (2019). Tibiotalocalcaneal arthrodesis using a novel retrograde intramedullary nail. Foot Ankle Spec..

[23] Rammelt S (2013). Tibiotalocalcaneal fusion using the hindfoot arthrodesis nail: a multicenter study. Foot Ankle Int..

[24] Biz C, Hoxhaj B, Aldegheri R, Iacobellis C (2016). Minimally invasive surgery for tibiotalocalcaneal arthrodesis using a retrograde intramedullary nail: preliminary results of an innovative modified technique. J. Foot Ankle Surg..

